# Immunohistochemistry and Bioinformatics Identify GPX8 as a Potential Prognostic Biomarker and Target in Human Gastric Cancer

**DOI:** 10.3389/fonc.2022.878546

**Published:** 2022-05-27

**Authors:** Xiaojie Zhang, Heng Xu, Yunan Zhang, Chongyuan Sun, Zefeng Li, Chunfang Hu, Dongbing Zhao, Chunguang Guo

**Affiliations:** ^1^ Department of Pancreatic and Gastric Surgical Oncology, National Cancer Center/National Clinical Research for Cancer/Cancer Hospital, Chinese Academy of Medical Sciences and Peking Union Medical College, Beijing, China; ^2^ School of Life Sciences, Beijing University of Chinese Medicine, Beijing, China; ^3^ Department of Pathology, National Cancer Center/National Clinical Research Center for Cancer/Cancer Hospital, Chinese Academy of Medical Sciences and Peking Union Medical College, Beijing, China

**Keywords:** GP X8, stomach cancer, bioinformatics, prognosis, immunohistochemistry

## Abstract

**Background:**

Glutathione peroxidase 8 (GPX8) is a type II transmembrane protein with rare structural features belonging to the glutathione peroxidase family. The function of GPX8 in stomach adenocarcinoma has not been discovered clearly.

**Methods:**

In this study, we comprehensively analyzed the expression of GPX8 in stomach adenocarcinoma and discovered that it is a potential target in the treatment of stomach adenocarcinoma. The immunohistochemical staining of GPX8 and survival analysis were performed in carcinoma tissue and adjacent tissues of 83 gastric cancer patients. The Gene Expression Profiling Interactive Analysis (GEPIA) database and Kaplan–Meier plotter database were used to evaluate the prognostic survival of GPX8 in stomach adenocarcinoma. The Cancer Genome Atlas (TCGA) database was used to download the microarray mRNA data of GPX8 and clinical information for cancer patients. The TIMER database and GSEA database were used to systematically evaluate the association of GPX8 and tumor-infiltrating lymphocytes in adenocarcinoma carcinoma. The STRING database was used to analyze protein-to-protein interactions of GPX8. The ROC curve was used to analyze the diagnostic effect of GPX8 in distinguishing outcomes between different subgroups, and a nomogram was constructed based on GPX8. Top transcription factor binding sites were analyzed using the QIAGEN database in the GPX8 gene promoter, and the functional enrichment analysis of GPX8 was done by GO and KEGG pathway enrichment analyses.

**Result:**

Based on the GEPIA and TCGA databases, the mRNA expression of GPX8 was significantly higher in stomach adenocarcinoma compared with the adjacent normal tissues. The GEPIA and Kaplan–Meier plotter databases showed that a higher GPX8 expression level was correlated with poor prognosis of stomach adenocarcinoma, suggesting that GPX8 was a risk factor of poor prognosis in stomach adenocarcinoma. The TIMER database showed that the GPX8 expression level was positively correlated with infiltrating levels of CD8+ T cells, CD4+ T cells, macrophages, neutrophils, and dendritic cells in stomach adenocarcinoma. The GSEA database indicated that GPX8 was positively correlated with B cells, dendritic cells, CD4+ T cells, CD8+ T cells, macrophages, mast cells, monocytes, and natural killer cells. At last, GO analysis indicated that the biological processes were enriched in collagen fibril organization, endodermal cell differentiation, collagen metabolic process, extracellular matrix organization, etc. KEGG signaling pathway analysis showed that GPX8 was correlated with protein digestion and absorption, extracellular matrix receptor interaction, AGE/RAGE signaling pathway, etc. The GSEA database showed that GPX8 was positively associated with angiogenesis, epithelial mesenchymal transition, hedgehog signaling, etc. The immunohistochemical staining of GPX8 and survival analysis in 83 gastric cancer patients showed that the OS rate of patients with a high GPX8 expression was significantly lower than that of the low GPX8 expression group.

**Conclusion:**

GPX8 is an important factor which might be a potential target in the treatment of stomach adenocarcinoma.

## Introduction

Stomach cancer is one of the leading causes of death worldwide owing to cancer, which accounts for its being the second leading cause of cancer death and the fourth most common cancer (1). It is estimated that almost 950,000 new patients are diagnosed as stomach cancer every year worldwide. Although the incidence and mortality have decreased in recent years especially in USA and western Europe, the burdens of stomach cancer in northeast Asia, eastern Europe, and Latin America remain high (2). The traditional treatment methods of stomach cancer include surgical resection, chemotherapy, and radiation (3). However, the traditional treatment strategies of stomach cancer remain with some shortcomings. In recent years, targeted therapy based on a molecular subtype of stomach cancer has been the optimistic option to deal with stomach cancer. Trastuzumab, known as the monoclonal antibody against HER2, could prolong the progression-free survival (PFS) and overall survival (OS) in HER2-positive stomach cancer patients. Thus, it is important to uncover the molecular nature of stomach and develop new drugs to improve individual benefits.

Glutathione peroxidase 8 (GPX8), also named probable glutathione peroxidase 8, is a type II transmembrane protein with rare structural features belonging to the glutathione peroxidase family in amphibians and Mammalia (4). GPX8 has a C-terminal endoplasmic reticulum signal and an N-terminal signal peptide. Therefore, it is also an endoplasmic reticulum membrane protein. The function of GPX8 has not been discovered clearly. Mehmeti et al. found that GPX8 was associated with hydrogen peroxide generation, endoplasmic reticulum stress, and apoptosis induction (5). GPX8 could scavenge hydrogen peroxide production, reducing lethal oxidative stress and fueling disulfide bond formation. Yoboue et al. demonstrated that GPX8 was important in regulating Ca^2+^ homeostasis and signaling (6). GPX8 reduced Ca^2+^ and IP3-dependent cytosolic and mitochondrial Ca^2+^ transients. The conserved transmembrane domain of GPX8 regulates Ca^2+^ and redox signals at the interface of the mitochondria endoplasmic reticulum. However, the functions of GPX8 in cancers have not been explored.

In this study, we aim to discover the correlation between GPX8 and stomach adenocarcinoma. Firstly, the Gene Expression Profiling Interactive Analysis (GEPIA) and The Cancer Genome Atlas (TCGA) datasets were used to analyze the expression of GPX8 in stomach adenocarcinoma and adjacent normal tissues. Secondly, the GEPIA and Kaplan–Meier plotter databases were conducted to discover the prognostic role of GPX8 in stomach adenocarcinoma. The STRING dataset was used to investigate the GPX8 network of the protein–protein interaction. Then, we used the TIMER database to find the association between GPX8 and tumor-infiltrating lymphocytes in the tumor microenvironment. Gene Set Enrichment Analysis (GSEA) was performed to reveal the biological functions including immune cells and signaling pathways of GPX8 in stomach adenocarcinoma. At last, Gene Ontology (GO) and Kyoto Encyclopedia of Genes and Genomes (KEGG) pathway analyses were used to demonstrate the biological functions of GPX8 in stomach adenocarcinoma. We hope to identify the function of GPX8 in stomach adenocarcinoma with this research.

## Methods

### GEPIA Database

In this study, we used the Gene Expression Profiling Interactive Analysis (GEPIA, http://gepia.cancerpku.cn/) database (7) to discover the difference in the GPX8 expression between stomach adenocarcinoma and adjacent normal tissue. GEPIA is a powerful bioinformatic database based on TCGA and genotype-tissue expression (GTEx) data, which has processed more than 280,000 analysis requests for 110,000 users in the recent 2 years. It includes the expression of RNA sequencing data of 9,736 tumor tissues and 8,587 normal tissues. The sample number of stomach adenocarcinoma in the GEPIA database was 408, while the sample number of normal tissue was 211. In addition, the GEPIA database was used to evaluate the prognostic role of GPX8 in stomach adenocarcinoma in this study.

### TCGA Database

The Cancer Genome Atlas (TCGA, https://portal.gdc.cancer.gov) was used to download the microarray mRNA data of GPX8. Clinical information from 443 patients with gastric cancer and gene expression data from 375 gastric cancer patients (workflow type: HTSeq-Count) were acquired by using TCGA database. GPX8 mRNA expression was analyzed between stomach adenocarcinoma and normal adjacent tissues. SPSS 20.0 was conducted to calculate the statistical significance.

### Kaplan–Meier Plotter Database

The Kaplan–Meier Plotter database (http://kmplot.com/analysis/) is a useful dataset platform to analyze the GPX8 expression on the prognostic potential role of stomach adenocarcinoma. Gene expression data and relapse-free and overall survival information in the Kaplan–Meier plotter database (8) are downloaded from GEO, EGA, and TCGA. In this study, we assessed the influence of GPX8 on survival in stomach adenocarcinoma patients. The 95% confidence interval hazard ratio and log-rank *P* value were evaluated.

### TIMER Database

The TIMER database (http://cistrome.org/TIMER/) is a powerful web resource to systematically evaluate the diverse immune cells’ impact in different types of cancer. The RNA-seq data of the TIMER database come from TCGA database (9). In this study, we analyzed the correlation between GPX8 and immune cells’ status in the tumor microenvironment of stomach adenocarcinoma. Purity, B cells, CD4^+^ T cells, CD8^+^ T cells, neutrophils, macrophages, and dendritic cells were estimated in the tumor microenvironment.

### STRING Database

The Search Tool for the Retrieval of Interacting Genes (STRING, https://string-db.org/) database (10) was conducted to evaluate the protein–protein interaction (PPI) network of GPX8. Number of edges, number of nodes, value of PPI enrichment, and degree of average node could be analyzed using the STRING database. Nodes were classified by biological processes. Edges were directed by molecular functions. The connectivity degrees of nodes were evaluated in the PPI network.

### GSEA Database and QIAGEN Database

Gene Set Enrichment Analysis (GSEA, https://www.gsea-msigdb.org/gsea/msigdb/index.jsp) (11) was used to investigate the biological functions of GPX8 in stomach adenocarcinoma. It is a powerful bioinformatics dataset to discover the classes of genes which are overrepresented in large sets of genes and analyze the correlation in disease phenotypes. It could assess the statistical significance of genes in different biological states. Important factors including enrichment score and multiple-hypothesis testing adjustment are calculated in the GSEA dataset. The gene set would show to be positively enriched if it is highly expressed with a high risk score. The *P*-value of a significant gene is regarded as <0.05, while the significance of the false discovery rate (FDR) is considered as <0.25. Top transcription factor binding sites were analyzed by QIAGEN (https://geneglobe.qiagen.com/cn/analyze) in the GPX8 gene promoter.

### GO Analysis and KEGG Analysis

Gene Ontology (GO) analysis is a useful bioinformatics method to investigate the biological processes, molecular functions, and cellular components of the selected gene. Biological processes combine functions of living units including cells, tissues, organ, and organisms. It represents specific objectives that living units are genetically programmed to achieve. Molecular functions occur at a single macromolecular machine in the elemental activities. The molecular functions in GO analysis represent gene product activities to perform actions. Cellular components indicate the cellular function occupied by a macromolecular machine. In this study, we evaluated the biological processes with GO analysis.

Kyoto Encyclopedia of Genes and Genomes (KEGG) analysis contains multiple database collections such as chemical substances, genomes, and biological pathways. KEGG could compare genome maps, browse genome maps, and manipulate expression maps with Java tools. It integrates the items containing metabolism, cell cycle, membrane transport, and signal transduction. KEGG also could conducts graph comparison, sequence comparison, and path computation.

### GPX8 Immunohistochemistry in the Gastric Cancer Tissue Array

GPX8 immunohistochemistry was performed in the gastric cancer tissue array. The tissue array was provided by Shanghai Tufei Biology and Technology Co., Ltd. The tissue chip was composed of 166 sample spots, including 83 cases with paired tumor and adjacent normal samples.

The tissue array was sectioned into 5-μm thickness for subsequent immunohistochemical staining. Briefly, after deparaffinization, endogenous peroxidases were blocked for 20 min in 3% of H_2_O_2_. Antigen retrieval was performed by heating the samples in 10 mmol/l citrate buffer (pH 6.0) at 95°C for 60 min. Then, sections were incubated at 4°C overnight with an anti-GPX8 antibody (Abcam, Cambridge, MA, USA) at a dilution of 1:300. Immunohistochemical staining was visually graded according to the intensity of stained cells as low (+), moderate (++), or high (+++). Immunostaining was evaluated by two different pathologists. Three positions were selected for each tissue slice, and the average optical density values were measured respectively. Finally, the average value of the three average optical density values was used as an indicator to measure the expression of GPX8.

### Statistical Analysis

Data are presented as mean ± SD in the statistical analyses. The SPSS 20.0 software was used to measure the data. *P* < 0.05 was regarded as statistical significance.

### Ethics Approval and Consent to Participate

Written informed consent was obtained from each patient involved in the study. This work complied with the Declaration of Helsinki and was approved by the Ethics Committee of National Cancer Center, Cancer Hospital, Chinese Academy of Medical Sciences and Peking Union Medical College (Approval Number: NCC2016XQ-44).

## Results

### Expression and Distribution of the GPX8 Gene in Pan-Cancer Perspective and Stomach Adenocarcinoma

**Figure 1:**
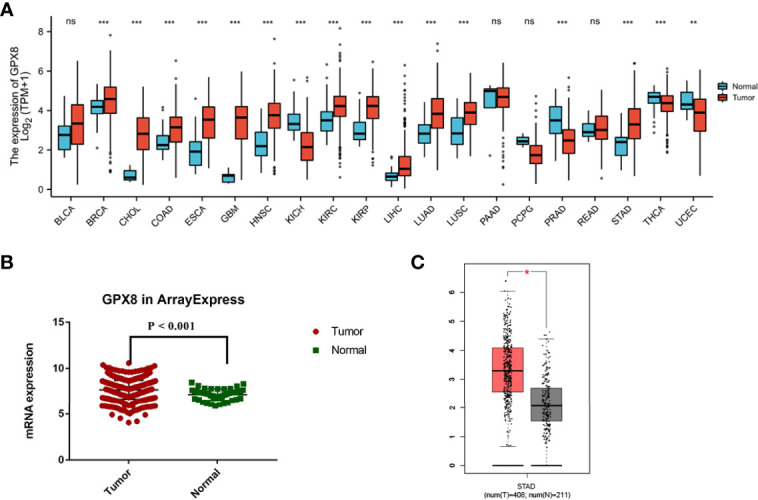
Expression pattern of GPX8 in pan-cancer perspective and stomach adenocarcinoma and ROC curve. **(A)** Expression pattern of GPX8 in pan-cancer perspective (ns, *P* ≥ 0.05; *, *P* < 0.05; **, *P* < 0.01; ***, *P* < 0.001). **(B)** In TCGA database, the mRNA expression of GPX8 in ArrayExpress was significantly higher in stomach adenocarcinoma (red) than the adjacent normal tissue (green) (P<0.01). **(C)** Differential expression analysis of GPX8 between adjacent normal tissue (gray) and stomach adenocarcinoma (red) in the GEPIA database.

Since the cancer types contained less than five normal group samples, the data sets of 15 cancer types were excluded from pan-cancer. Finally, we evaluated the mRNA expression pattern of GPX8 in the remaining 20 cancer types. As shown in **Figure 1A**, GPX8 was significantly upregulated in 12 of all 20 cancer types compared with normal tissues. These data suggest that the mRNA expression of GPX8 is abnormally expressed in different cancer types.

We investigated the expression of GPX8 between stomach adenocarcinoma and normal adjacent tissue with the GEPIA database. The sample number of stomach adenocarcinoma was 408, while the sample number of normal tissue was 211. We used TCGA database to validate the results. The mRNA expression of GPX8 in ArrayExpress was significantly higher in stomach adenocarcinoma than the adjacent normal tissue (**Figure 1B**). In addition, the result of the GEPIA database indicated that the mRNA expression of GPX8 in stomach adenocarcinoma was significantly higher than the adjacent normal tissue (*P*<0.05) (**Figure 1C**). Combining the results of the GEPIA and TCGA databases, the GPX8 expression in stomach adenocarcinoma was higher than that in normal tissue.

### The High Expression of GPX8 Is Closely Related to the Poor Prognosis of STAD and Subgroups of Patients

**Figure 2:**
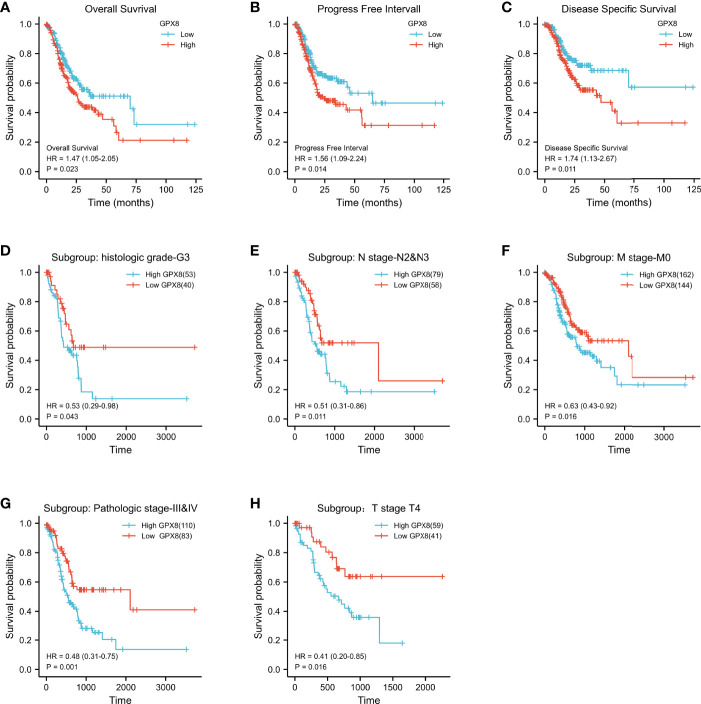
Kaplan–Meier survival curves comparing the high and low expressions of GPX8 in STAD. **(A–C)** Survival curves of OS, DSS, and PFI between GPX8-high and -low patients with STAD. **(D–H)** OS survival curves of grade G3, N2 and N3, M0, stage III and IV, and T4 subgroups between GPX8-high and -low patients with STAD (STAD, stomach adenocarcinoma; OS, overall survival; DSS, disease-specific survival; PFI, progression-free interval).

Patients with a high expression of GPX8 have lower 10-year OS rates than those with a low expression of GPX8 (HR = 1.47(1.05–2.05); *P* = 0.023; **Figure 2A**). Similarly, the 10-year PFI and DSS rates in the GPX8-high group were significantly lower than those in the GPX8-low group (HR = 1.56(1.09–2.24), *P* = 0.014; HR = 1.74(1.13–2.67), *P* = 0.011; **Figures 2B, C**).

Then, we evaluated the ability of high and low expressions of GPX8 to predict death in each subgroup. The results showed that the prognosis of patients with GPX8-high was poor in the histologic grade G3 (HR = 0.53, *P* = 0.043), N2 and N3 (HR = 0.51, *P* = 0.011), M0 (HR = 0.63, *P* = 0.016), stage III and IV (HR=0.48, *P* = 0.001), and T4 (HR=0.41, *P* = 0.016) subgroups of OS (**Figures 2D–H**). Subgroup analysis can further help us determine the predictive efficacy of GPX8 in specific populations, which indicates that a high expression of GPX8 represents a worse prognosis in the following patients: patients without distant metastasis, patients with tumor invading the serosal layer, or patients with poorly differentiated tissue.

### Association With GPX8 Expression and Clinicopathological Variables

To clarify the significance of the GPX8 expression, a total of 407 stomach cancer samples with GPX8 expression data were analyzed from TCGA. These samples have characteristics of all patients, and the clinical cohort included 241 men and 134 women.

**Figure 3:**
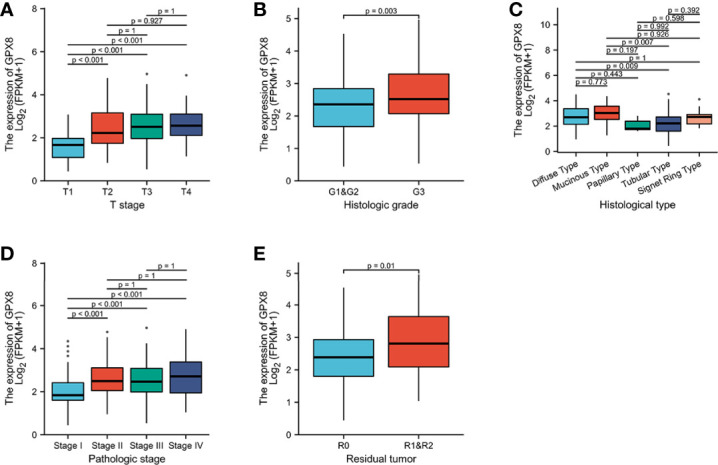
Association between GPX8 expression and clinicopathological characteristics, including **(A)** T stage, **(B)** histologic grade, **(C)** histological type, **(D)** pathologic stage, and **(E)** residual tumor.

As shown in **Figures 3A–E** and **Table S1**, the overexpression of GPX8 is significantly related to the clinical features of stomach cancer, such as histologic grade (G1 and G2 vs. G3, *P* = 0.003), pathologic stage (stage 1vs. stage 2, *P* < 0.001; stage 1 vs. stage 3, *P* < 0.001), T stage (T1 vs. T2, *P* < 0.001; T1 vs. T3, *P* < 0.001; T1 vs. T4, *P* < 0.001), and histological type (diffuse type (DT) vs. tubular type (TT), *P* = 0.009; mucinous type (MT) vs. tubular type (TT), *P* = 0.007). GPX8 expression has nothing to do with other clinicopathological characteristics (**Figures S1A–D**).

Univariate analysis of logistic regression showed that the expression of GPX8 as a categorical dependent variable was related to the clinicopathological characteristics of poor prognosis (**Table S2**). The enhanced expression of GPX8 in stomach cancer is positively correlated with the following clinical characteristics, such as T stage (OR = 2.032 for T1, T2 vs. T3, T4, *P* = 0.003), N stage (OR = 2.032 for N1, N2, and N3 vs. N0, *P* =0.018), and pathologic stage (OR = 3.495 for stage III, stage IV, and stage II vs. stage I, *P* < 0.001). These results indicate that compared with low GPX8 expression, stomach cancer patients with a high GPX8 expression are easier to have a higher stage.

### The ROC Curve Was Used to Analyze the Diagnostic Effect of GPX8 in Distinguishing Outcomes Between Different Subgroups

**Figure 4:**
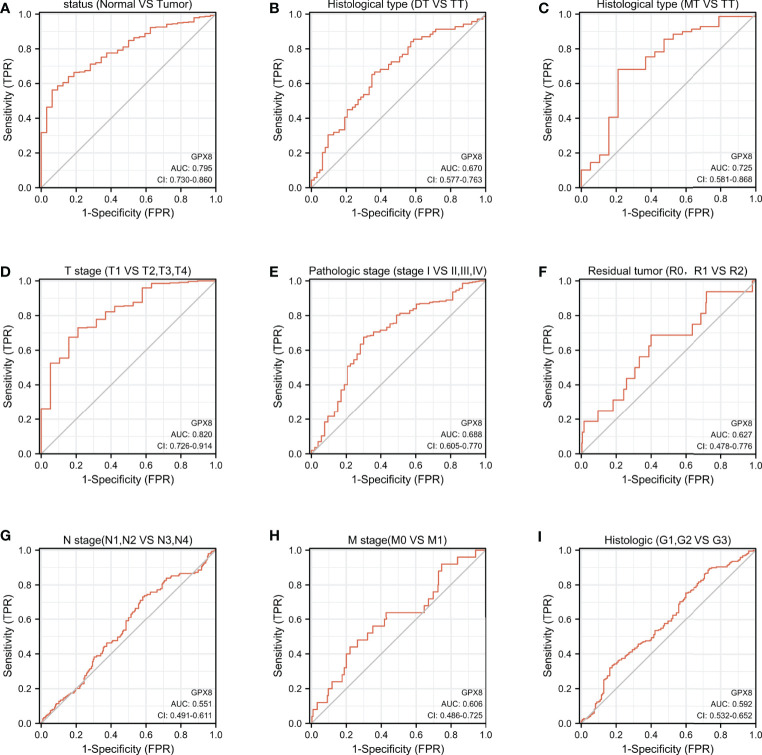
ROC curve was used to analyze the diagnostic effect of GPX8 in distinguishing outcomes between different subgroups. **(A)** status (Normal vs Tumor); **(B)** Histological type (DT vs TT); **(C)** Histological type (MT vs TT); **(D)** T stage (T1 vs T2, T3, T4); **(E)** Pathologic stage (stage I vs II, III, IV); **(F)** Residual tumor (R0, R1 vs R2); **(G)** N stage (N1, N2 vs N3, N4); **(H)** M stage (M0 vs M1); **(I)** Histologic (G1, G2 vs G3). Note: The value of the area under the ROC curve is between 0.5 and 1. The closer the AUC is to 1, the better the diagnostic effect. AUC has low accuracy when it is 0.5∼0.7, it has certain accuracy when AUC is 0.7∼0.9, and it has high accuracy when AUC is above 0.9. DT, Diffuse Type; TT, Tubular Type; MT, Mucinous Type; R0, no residual tumor; R1, microscopic residual tumor; R2, macroscopic residual tumor; G1, Grade 1; G2, Grade 2; G3, Grade 3.

It is shown in **Figures 4A, C, D** that GPX8 has certain accuracy in predicting outcomes between tumor and normal (AUC = 0.795, CI = 0.730–0.860), in predicting MT and TT outcomes (AUC = 0.725, CI = 0.581–0.868), and in predicting the outcome of T1, T2, T3, and T4 (AUC = 0.820, CI = 0.726–0.914). In addition, as shown in **Figures 4B, D–I**, GPX8 has lower accuracy for the prediction in the remaining subgroups.

### Top Transcription Factors Binding Sites by QIAGEN in the GPX8 Gene Promoter

**Figure 5:**
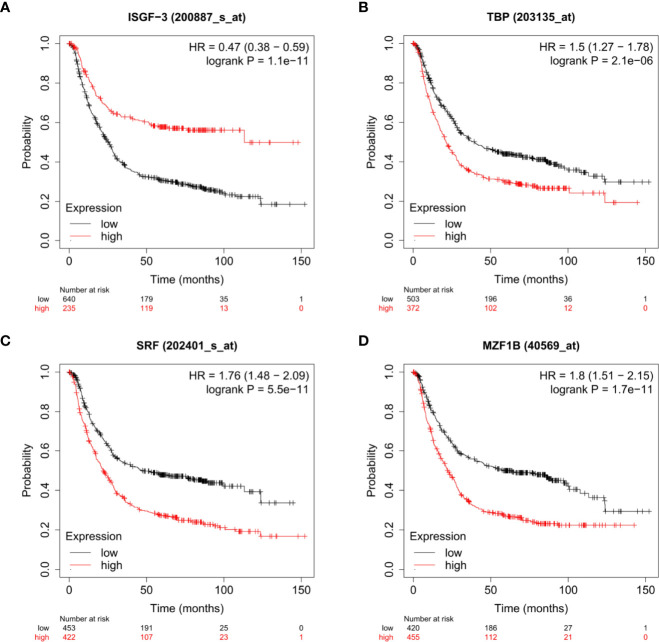
The relationship between the expressions of ISGF-3 **(A)**, TBP **(B)**, SRF **(C)**, and MZF1 **(D)** and the prognosis of stomach cancer patients were shown by Kaplan–Meier survival curves.

We found top transcription factor binding sites by QIAGEN in the GPX8 gene promoter, which contains ISGF-3, MZF1, SRF, and TBP. As shown in **Figure 5**, we analyzed the relationship between the expression of ISGF-3, MZF1, SRF, and TBP and the prognosis of stomach cancer patients by the Kaplan–Meier plotter database. The results show that the expression of ISGF-3, TBP, MZF1, and SRF is significantly related to the survival of stomach cancer patients (ISGF-3: HR = 0.47, log-rank *P* = 1.1e^-11^; TBP: HR = 1.76, log-rank *P* = 5.5e^-11^; SRF: HR = 1.76, log-rank *P* = 5.5e^-11^; MZF1B: HR = 1.8, log-rank *P* = 1.7e^-11^).

### Construction and Validation of a Nomogram on GPX8

As shown in **Table 1**, univariate Cox regression correlation analysis shows that these clinical factors are significantly related to overall survival, such as T stage, N stage, M stage, age, pathologic stage, primary therapy outcome, and GPX8. In multivariate Cox regression analysis, the factors (T stage, N stage, pathologic stage, histologic grade, primary therapy outcome, and GPX8) are related to overall survival rate. It can be seen that the high expression of GPX8 is still an independent factor significantly related to OS rate (HR = 1.753; CI = 1.223–2.514; *P* = 0.002).

**Table 1:** Univariate and multivariate COX regression in patients with stomach cancer.

Characteristics	Total (N)	Univariate analysis		Multivariate analysis	
		Hazard ratio (95% CI)	P value	Hazard ratio (95% CI)	P value
**T stage**	362				
T1 and T2	96	Reference			
T4 and T3	266	1.719 (1.131–2.612)	**0.011**	2.860 (1.011–8.091)	**0.048**
**N stage**	352				
N0	107	Reference			
N1 and N2 and N3	245	1.925 (1.264–2.931)	**0.002**	1.439 (0.499–4.150)	0.501
**Pathologic stage**	347				
Stage I	50	Reference			
Stage IV and stage III and stage II	297	2.247 (1.210–4.175)	**0.010**	0.298 (0.042–2.124)	0.227
**Histologic grade**	361				
G1 and G2	144	Reference			
G3	217	1.353 (0.957–1.914)	0.087	2.404 (1.005–5.748)	**0.049**
**Histological type**	156				
Diffuse type	63	Reference			
Mucinous type	19	0.288 (0.087–0.955)	**0.042**	0.575 (0.150–2.209)	0.420
Papillary type	5	1.635 (0.491–5.441)	0.423	12.720 (2.500–64.726)	**0.002**
Tubular type	69	0.950 (0.546–1.654)	0.856	1.467 (0.664–3.240)	0.343
**Primary therapy outcome**	313				
CR	229	Reference			
PD and SD and PR	84	4.228 (2.905–6.152)	**<0.001**	5.264 (2.612–10.610)	**<0.001**
**GPX8**	370	1.359 (1.128–1.638)	**0.001**	1.753 (1.223–2.514)	**0.002**
**STAT1**	370	0.980 (0.824–1.167)	0.823	1.085 (0.793–1.486)	0.608
**MZF1**	370	0.869 (0.612–1.235)	0.434	0.741 (0.380–1.444)	0.379
**SRF**	370	0.990 (0.755–1.299)	0.944	1.028 (0.621–1.701)	0.916
**TBP**	370	0.806 (0.504–1.288)	0.367	0.530 (0.172–1.637)	0.270

Bold font means P-value < 0.05.

**Figure 6:**
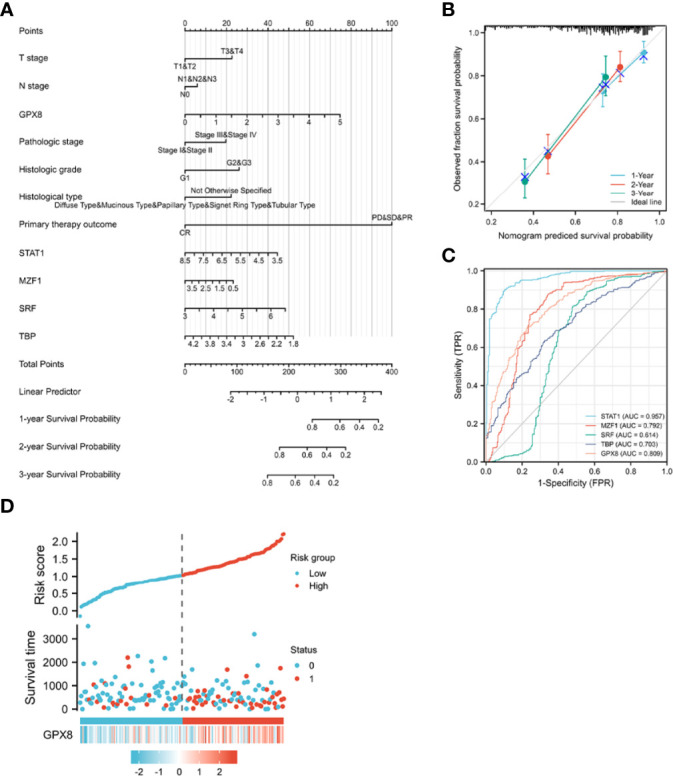
A quantitative method to predict the 1-, 2-, and 5- year OS in STAD patients. **(A)** A nomogram for predicting the probability of 1-, 3-, and 5- year OS for STAD patients. **(B)** Calibration plots of the nomogram for predicting the probability of OS at 1, 2, and 3 years. **(C)** The ROC curve of the GPX8 and its transcription factors in over-survival time. OS, overall survival. **(D)** GPX8 expression distribution and survival status.

To provide a quantitative method for predicting the prognosis of patients with stomach cancer, GPX8, transcription factors (ISGF-3, TBP, MZF1, SRF), and independent clinical risk factors were used to construct a nomogram (**Figure 6A**). On the basis of multivariate Cox regression analysis, a ruler score is set to characterize each variable in the multivariate regression model, and finally the total calculated score is used to predict the probability of event occurrence. The sum of the points assigned to each variable was adjusted to a range of 1 to 100. The scores of the variables were accumulated as the total score. The vertical line was used to find the corresponding results of the total score (1-year, 2-year, and 3-year survival probabilities). We further analyzed the prediction efficiency of the nomogram. The bias-corrected line in the calibration plot was utilized to be close to the ideal curve (the 45-degree line), which showed a fine agreement between the prediction and the observation (concordance, C-index= 0.759 (0.726–0.793); likelihood ratio test = 49.27 on 13 df, *P* = 3.97e^-06^; score (log-rank) test = 51.79 on 13 df, *P* = 1.46e^-06^; Wald test = 44.65 on 13 df, P = 2.39e^-05^) (**Figure 6B**).

Concerning distinguishing normal tissue and tumor, the predictive ability of STAT1 has a high accuracy (AUC = 0.957, CI = 0.942–0.973), the predictive ability of MZF1, GPX8, and TBP has a certain accuracy (AUC = 0.792, CI = 0.749–0.834; AUC = 0.809, CI = 0.774–0.845; AUC = 0.703, CI = 0.660–0.745), and the predictive ability of SRF has a lower accuracy (AUC = 0.614, CI = 0.558–0.669) (**Figure 6C**). The risk factor graph shows that the high expression of GPX8 is more related to the high risk of death (**Figure 6D**).

### The Association of GPX8 and Tumor-Infiltrating Lymphocytes in Adenocarcinoma Carcinoma

**Figure 7:**
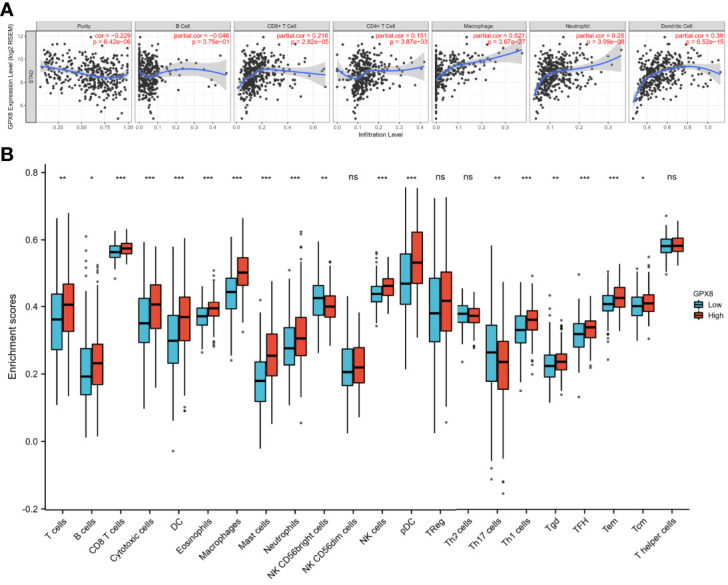
The association of GPX8 and tumor-infiltrating lymphocytes in adenocarcinoma carcinoma. **(A)** GPX8 was positively correlated with infiltrating levels of CD4+ T cells, CD8+ T cells, macrophages, neutrophils, and dendritic cells in stomach adenocarcinoma. GPX8 was negatively correlated with purity of immune cells and infiltrating levels of B cells. **(B)** The varied proportions of 22 subtypes of immune cells in the high and low GPX8 expression groups in tumor samples. ns, p ≤0.05; *, p < 0.05; **, p < 0.01; ***, p< 0.001.

We used the TIMER database to discover the association of GPX8 and tumor-infiltrating lymphocytes in stomach adenocarcinoma (**Figure 7A**). The results showed that the GPX8 expression level was positively correlated with infiltrating levels of CD8+ T cells (r = 0.216, *P* = 2.82e−05), CD4+ T cells (r = 0.151, *P* = 3.87e−03), macrophages (r = 0.521, *P* = 3.67e−27), neutrophils (r = 0.28, *P* = 3.99e−08), and dendritic cells (r = 0.39, *P* = 6.52e−15) in stomach adenocarcinoma. On the contrary, GPX8 expression was negatively correlated with purity and B-cell infiltration (r = -0.046, *P* = 3.75e−01).

We also used the ssGSEA algorithm to calculate the correlation between GPX8 and 24 immune cells. It can be seen from **Figure 7B** that patients with high GPX8 expression have a high degree of infiltration of various immune cells (CD8+ T cells, DC, Neutrophils, Macrophages, NK cells, etc.). It also can be seen from **Table 2** that the expression of GPX8 is significantly positively correlated with the degree of infiltration of various immune cells (DC, CD8+ T cells, Macrophages, Mast cells, NK cells).

**Table 2:** Correlation between GPX8 expression and immune cell infiltration.

Gene	Immune cells	Correlation coefficient (Pearson)	*P* (Pearson)	Correlation coefficient (Spearman)	*P* (Spearman)
GPX8	aDC	0.117	**0.023**	0.119	**0.022**
GPX8	B cells	0.122	**0.018**	0.146	**0.005**
GPX8	CD8 T cells	0.296	**<0.001**	0.291	**<0.001**
GPX8	Cytotoxic cells	0.243	**<0.001**	0.234	**<0.001**
GPX8	DC	0.357	**<0.001**	0.341	**<0.001**
GPX8	Eosinophils	0.322	**<0.001**	0.322	**<0.001**
GPX8	iDC	0.475	**<0.001**	0.451	**<0.001**
GPX8	Macrophages	0.555	**<0.001**	0.534	**<0.001**
GPX8	Mast cells	0.433	**<0.001**	0.419	**<0.001**
GPX8	Neutrophils	0.295	**<0.001**	0.229	**<0.001**
GPX8	NK CD56bright cells	-0.154	**0.003**	-0.175	**<0.001**
GPX8	NK CD56dim cells	0.120	**0.020**	0.111	**0.032**
GPX8	NK cells	0.353	**<0.001**	0.371	**<0.001**
GPX8	pDC	0.323	**<0.001**	0.326	**<0.001**
GPX8	T cells	0.198	**<0.001**	0.201	**<0.001**
GPX8	T helper cells	0.064	0.218	0.055	0.288
GPX8	Tcm	0.133	**0.010**	0.150	**0.004**
GPX8	Tem	0.329	**<0.001**	0.307	**<0.001**
GPX8	TFH	0.220	**<0.001**	0.216	**<0.001**
GPX8	Tgd	0.141	**0.006**	0.191	**<0.001**
GPX8	Th1 cells	0.348	**<0.001**	0.333	**<0.001**
GPX8	Th17 cells	-0.152	**0.003**	-0.178	**<0.001**
GPX8	Th2 cells	-0.040	0.445	-0.074	0.150
GPX8	TReg	0.125	**0.016**	0.120	**0.020**

Bold font means P-value < 0.05.

**Figure 8:**
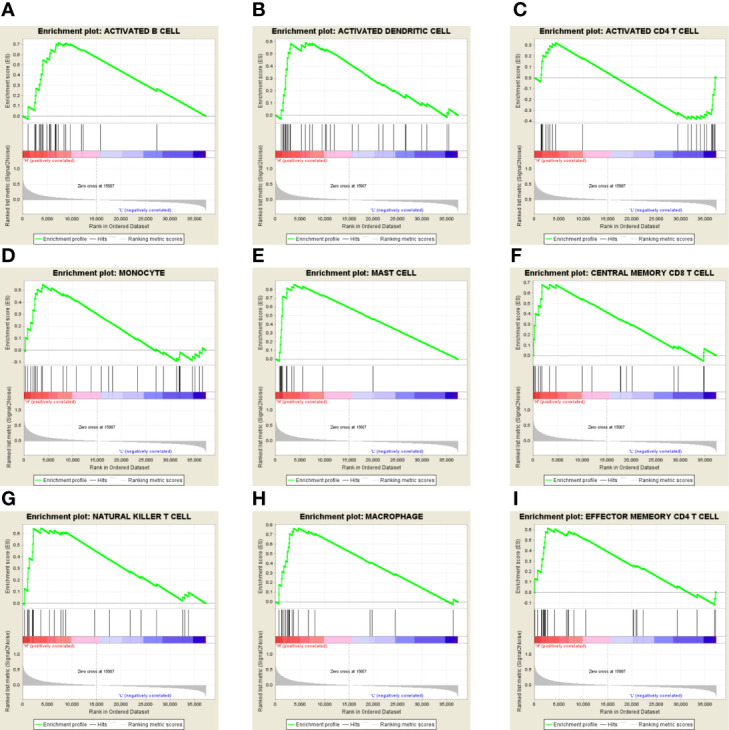
**(A–I)** In the GSEA database, GPX8 was positively correlated with B cells, dendritic cells, CD4+ T cells, CD8+ T cells, macrophages, mast cells, monocytes, natural killer cells, etc.

In addition, we also used the GSEA database to investigate the correlation between GPX8 and immune cells’ status in the tumor microenvironment of stomach adenocarcinoma. As shown in **Figures 8A–I**, GPX8 was positively correlated with B cells, dendritic cells, CD4+ T cells, CD8+ T cells, macrophages, mast cells, monocytes, natural killer cells, etc. The results indicated that GPX8 plays critical roles in the infiltration of tumor-infiltrating lymphocytes in the tumor microenvironment of stomach adenocarcinoma.

### Protein to Protein Interactions of GPX8

**Figure 9:**
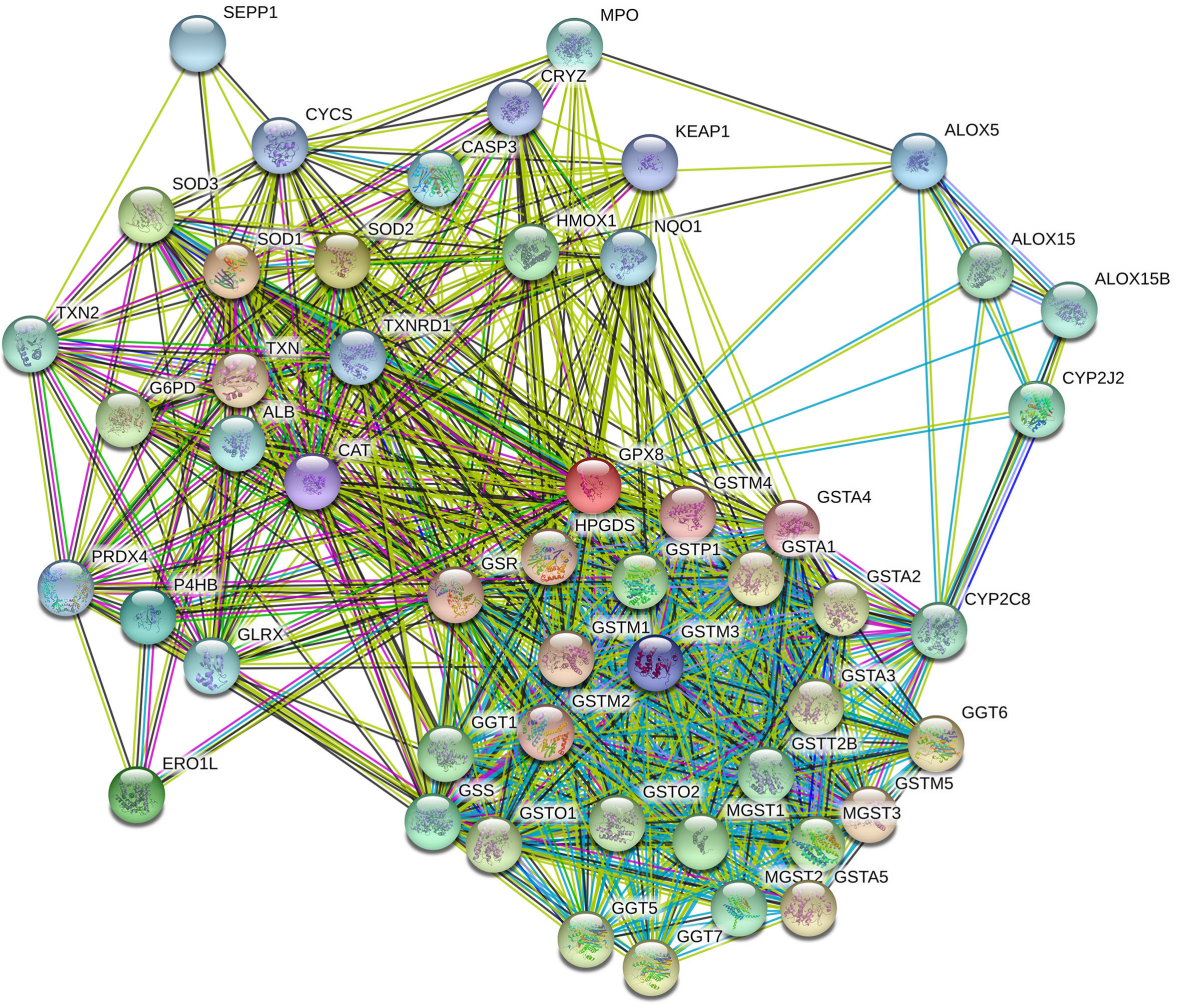
PPI network complex of GPX8. The number of nodes was 11, while the number of edges was 32. The average node degree was 5.82. The average local clustering coefficient was 0.858. The PPI enrichment P-value was 5.92e–07. The most significant nodes were SOD1, SOD2, SOD3, ERO1L, GSS, P4HB, ALOX5, GSTM3, CAT, and GSTA4.

We used STRING to explore the protein–protein interactions (PPI) related to GPX8 (**Figure 9**). There were 11 nodes and 32 edges in the complex PPI network. The edges mean the association of protein and protein, while the nodes mean the protein. In the PPI network, the most significant nodes were superoxide dismutase 1 (SOD1), SOD2, SOD3, ERO1-like protein alpha (ERO1L), glutathione synthetase (GSS), protein disulfide-isomerase (P4HB), arachidonate 5-lipoxygenase (ALOX5), glutathione S-transferase M3 (GSTM3), catalase (CAT), and glutathione S-transferase A4 (GSTA4).

### Functional Enrichment Analysis

**Figure 10:**
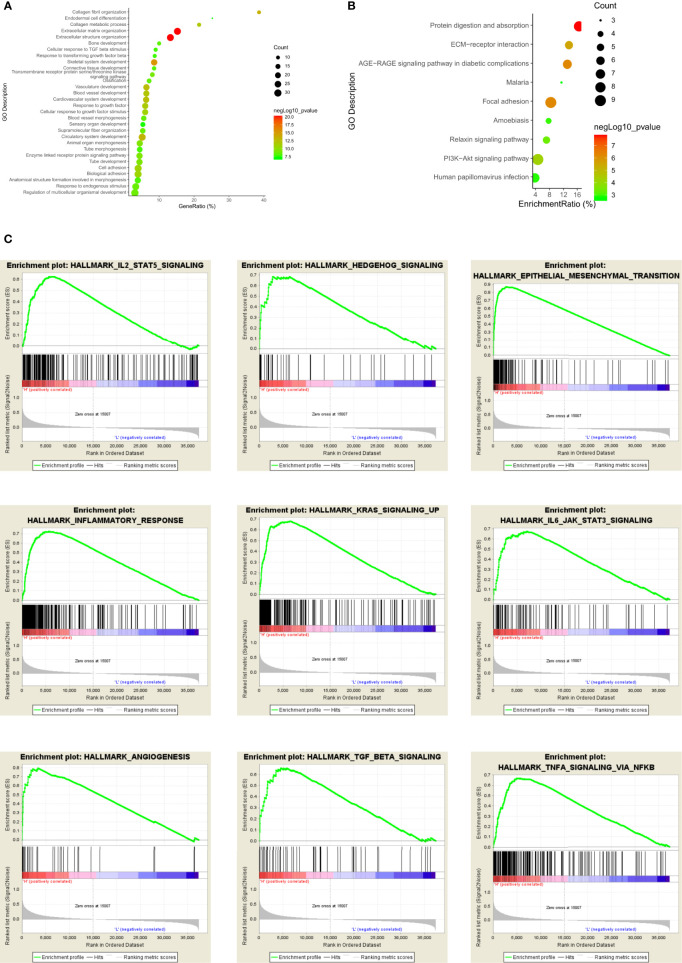
Functional enrichment analysis of GPX8 in stomach adenocarcinoma. **(A)** GO analysis of GPX8 in stomach adenocarcinoma. **(B)** KEGG signaling pathway of GPX8 in stomach adenocarcinoma. **(C)** GSEA analysis of GPX8 in stomach adenocarcinoma.

We conducted Gene Ontology (GO) analysis and Kyoto Encyclopedia of Genes and Genomes (KEGG) analysis to explore the biological signatures of GPX8 in stomach adenocarcinoma. GO analysis indicated that the biological processes were enriched in collagen fibril organization, endodermal cell differentiation, collagen metabolic process, extracellular matrix organization, extracellular structure organization, etc. (**Figure 10A**). KEGG signaling pathway analysis showed that GPX8 was correlated with protein digestion and absorption, extracellular matrix receptor interaction, AGE/RAGE signaling pathway, focal adhesion, relaxin signaling pathway, and PI3K/AKT signaling pathway (**Figure 10B**).

Furthermore, we used the GSEA database to validate the results of GO and KEGG analyses. The results showed that GPX8 was positively associated with angiogenesis, epithelial–mesenchymal transition, hedgehog signaling, IL2-STAT5 signaling pathway, IL6-JAK-STAT3 signaling pathway, inflammatory response, KRAS signaling pathway, etc (**Figure 10C**).

### Immunohistochemistry

**Figure 11:**
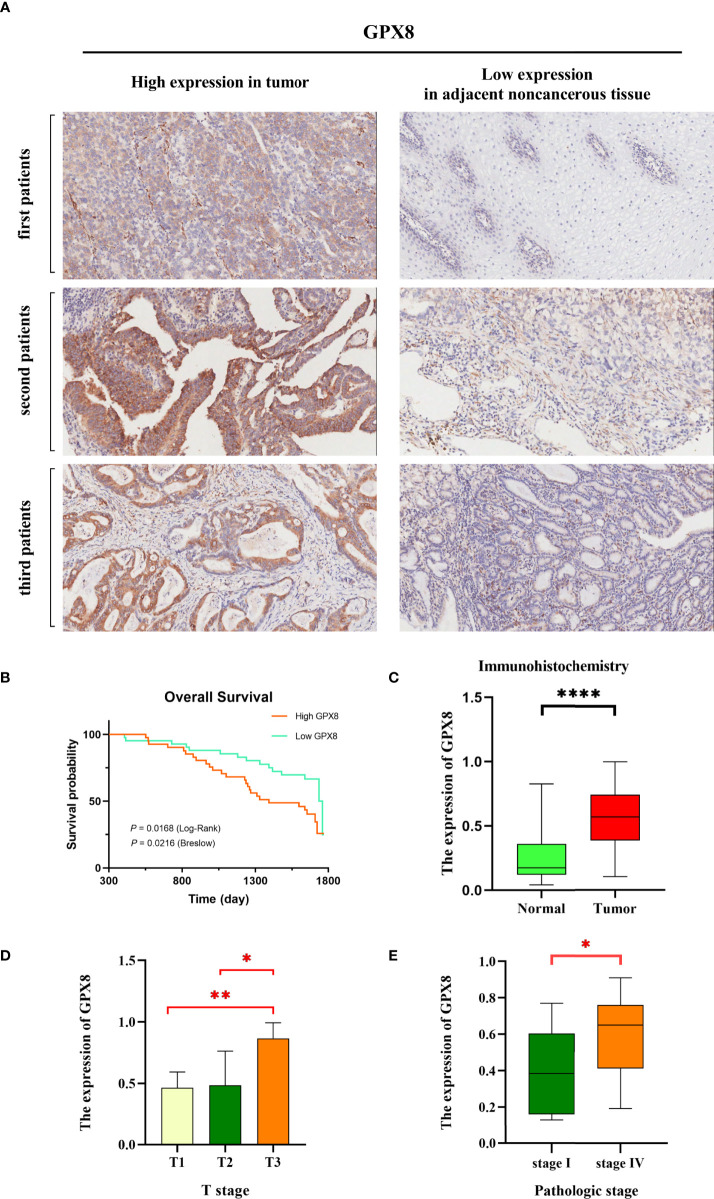
GPX8 gene expression levels were associated with clinical outcomes. **(A)** We selected six representative immunohistochemical pictures to show the expression of GPX8 in tumor and adjacent noncancerous tissues. **(B)** The Kaplan–Meier curve showed that a low GPX8 expression was associated with higher OS in stomach cancer patients, and the *P* value was calculated by log-rank (*P* = 0.0168) and Breslow (*P* = 0.0216). **(C)** The expression of GPX8 in tumor is higher than that in normal tissues. **(D, E)** There is a significant difference in the level of GPX8 expression between different groups, such as T1 vs. T3 (*P* = 0.0091) in the T stage, T2 vs. T3 (*P* = 0.0451) in the T stage, and stage I vs. stage IV (*P* = 0.0416) in the pathologic stage. *, p < 0.05; **, p < 0.01; ****, p < 0.0001.

In our study, we used immunohistochemistry to determine the expression level of GPX8 in cancer tissues and adjacent normal tissues of 83 patients. The immunohistochemical results of tumor and adjacent non-cancerous tissues in three patients are shown in **Figures 11A, C**; the expression of GPX8 in cancer tissues was significantly higher than that in adjacent normal tissues. In addition, according to the median of GPX8 expression in cancer tissues, 83 patients were divided into the high GPX8 expression group and low GPX8 expression group. The OS rate of patients with a high GPX8 expression was significantly lower than that of the low GPX8 expression group (*P* = 0.0168 (log-rank), **Figure 11B**). Finally, we analyzed the correlation between the clinical characteristics of 83 patients and the expression level of GPX8, and the results are shown in **Figures 11D, E**. The clinical characteristics of patients (T stage and pathologic stage) have a certain correlation with the level of GPX8. There is a significant difference in the level of GPX8 expression between different groups, such as T1 vs. T3 (*P* = 0.0091) in the T stage, T2 vs. T3 (*P* = 0.0451) in the T stage, and stage I vs. stage IV (*P* = 0.0416) in the pathologic stage. Therefore, the results of immunohistochemistry validate and support the previous prediction.

## Discussion

GPX8 belongs to the family of glutathione peroxidases. Eight glutathione peroxidases (GPX1-GPX8) have been identified so far in mammals. The glutathione peroxidase family takes part in the homeostasis of H_2_O_2_ in signaling pathways. Previous studies have demonstrated that GPX1 to GPX7 play important roles in the carcinogenesis of tumor. GPX1 could prevent carcinogenesis by reducing the mutation of oxidative DNA and diminishing the production of inflammatory cytokines such as leukotrienes and prostaglandins (12). The overexpression of GPX1 could reduce the tumor growth *in vivo* and *in vitro* (13). The level of GPX2 was increased in lung adenocarcinoma and colorectal cancer (14). GPX3 was considered as a novel tumor suppressor which is downregulated in several types of cancer (15, 16). GPX4 decreased the hydroperoxides in membranes to interfere the membrane oxidation (17). GPX6 increased an adaptive response to a more oxidized environment (18). GPX7 was involved in the protein folding in the endoplasmic reticulum (19). GPX8 was a novel member in the glutathione peroxidase family. The functions of GPX8 in cancers have not been investigated clearly. Thus, we aimed to discover the function of GPX8 in stomach adenocarcinoma in this study.

A recent article also found that GPX8 can significantly predict prognosis in gastric cancer (20). They found that aberrant GPX8 expression is associated with clinical features of gastric cancer, such as T stage, clinical stage, histological grade, residual tumor status, ethnicity, and patient survival. It was also found that the differential genes between patients with high and low expressions of GPX8 were significantly enriched in gene sets such as KEGG_BASAL_CELL_CARCINOMA by GSEA. In our work, we assessed the association of GPX8 and tumor-infiltrating lymphocytes in adenocarcinoma carcinoma. We also analyzed the diagnostic effect of GPX8 in distinguishing outcomes between different subgroups by the ROC curve. This helps doctors to more accurately diagnose the prognosis of patients with different clinical characteristics through the expression of GPX8. Finally, we constructed a clinical prognostic model based on clinical characteristics and GPX8, which could accurately predict the mortality of gastric cancer patients at 1, 2, and 3 years. In addition, we validated the correlation of the differential expression of GPX8 with clinical features and prognosis of gastric cancer patients by collecting clinical samples from hospitals.

Specifically, we discovered the mRNA expression of GPX8 in stomach adenocarcinoma and normal adjacent tissue with the GEPIA database and TCGA database. The GEPIA database showed that GPX8 was significantly higher in stomach adenocarcinoma than the adjacent normal tissues. TCGA database also indicated that GPX8 was significantly higher in stomach adenocarcinoma than the adjacent normal tissue. These results implied that GPX8 contributes to the carcinogenesis of stomach adenocarcinoma. Then, we discovered the association between GPX8 and prognosis of stomach adenocarcinoma with the GEPIA and Kaplan–Meier plotter databases. A higher GPX8 expression level was correlated with poor survival of stomach adenocarcinoma. This suggested that GPX8 might be a risk factor of dismal outcome.

After analyzing the mRNA expression and prognosis survival of GPX8 in stomach adenocarcinoma, we intended to investigate the correlation between the GPX8 expression level and immune status in the tumor microenvironment of stomach adenocarcinoma. The TIMER database showed that the GPX8 expression level was positively correlated with infiltrating levels of CD8^+^ T cells, CD4^+^ T cells, macrophages, neutrophils, and dendritic cells in stomach adenocarcinoma. The GSEA database indicated that GPX8 was positively correlated with B cells, dendritic cells, CD4^+^ T cells, CD8^+^ T cells, macrophages, mast cells, monocytes, and natural killer cells. Increased inflammatory cells could produce and secrete various types of chemokines and cytokines. These inflammatory mediators could recruit or traffic more types of inflammatory cells to the tumor microenvironment which exacerbates the vicious cycle. Therefore, GPX8 might exacerbate the stomach adenocarcinoma by enhancing the inflammation of tumor microenvironment.

Then, we assumed the PPI of GPX8 with the STRING dataset. The most significant nodes were SOD1, SOD2, SOD3, ERO1L, GSS, P4HB, ALOX5, GSTM3, CAT, and GSTA4. SOD1–3 are related to oxidative stress and reactive oxygen species in the tumor microenvironment (21). ERO1L is correlated with reactive oxygen species in the endoplasmic reticulum (22). GSS and P4HB are associated with oxidation and redox. ALOX5 belongs to the lipoxygenase family which contributes to innate immunity by regulating inflammatory responses (23). GSTM3 links to several types of cancers (24). CAT is also related to the reactive oxygen species and oxidative damage. GSTA4 is correlated with oxidative metabolism in several diseases including atherosclerosis, Alzheimer’s disease, and cancers (25). In brief, GPX8 interacts with the proteins which are focused on oxidative stress and reactive oxygen species.

Additionally, we conducted GO, KEGG, and GSEA databases to analyze the biological signatures of GPX8 in stomach adenocarcinoma. GO analysis indicated that the biological processes were enriched in collagen fibril organization, endodermal cell differentiation, collagen metabolic process, extracellular matrix organization, and extracellular structure organization. The extracellular matrix in the tumor microenvironment affects the growth and invasion of tumor. Proteins of the extracellular matrix construct the biochemical and physical niche of cancer stem cells (26). As the main component of the extracellular matrix, collagen in the microenvironment of tumor might affect the carcinogenesis and progression of cancers. In stomach cancer, collagen deposition would increase the incidence of stomach cancer (27). Enhanced collagen deposition and increased collagen width change the morphology of collagen fibers. The width of collagen is associated with prognostic survival in that the increased collagen width decreased the overall survival of stomach cancer patients. In our study, collagen fibril organization, collagen metabolic process, extracellular matrix organization, and extracellular structure organization are shown in GO analysis. Therefore, GPX8 might increase carcinogenesis of stomach adenocarcinoma by modulating collagen fibril and the extracellular matrix.

KEGG signaling pathway analysis showed that GPX8 was correlated with protein digestion and absorption, extracellular matrix receptor interaction, AGE/RAGE signaling pathway, focal adhesion, relaxin signaling pathway, and PI3K/AKT signaling pathway. Extracellular matrix receptor interaction, protein digestion and absorption, and focal adhesion in KEGG analysis might be correlated with collagen fibril and extracellular matrix modulation in GO analysis. The AGE/RAGE signaling pathway activates other signaling pathways such as Janus kinase/signal transducers and activators of transcription (JAK/STAT), mitogen-activated protein kinase (MAPK), and nuclear factor kappa-light-chain-enhancer of activated B cells (NF-κB) pathways (28). These signaling pathways take part in the proliferation and inflammation in cancers. The relaxin signaling pathway influences the growth and differentiation of tumor cells. Relaxin increases the expression of the vascular endothelial growth factor to stimulate the neovascularization and angiogenesis (29). Furthermore, relaxin promotes the invasion, attachment, and migration of tumor cells. The PI3K/AKT signaling pathway is important in regulating tumor cell functions such as metabolism, angiogenesis, and proliferation. Dysregulation of the PI3K/AKT signaling pathway exacerbates stomach cancers that it is regarded as the targeted therapy in stomach cancer (30). Therefore, apart from the validation of GO analysis, the results of KEGG signaling analysis also showed that some signaling pathways such as AGE/RAGE, relaxin, and PI3K/AKT signaling pathways were involved in the functions of GPX8 in stomach cancer.

At last, we used the GSEA database to validate the previous results. GPX8 was positively associated with angiogenesis, epithelial–mesenchymal transition, hedgehog signaling, IL6-JAK-STAT3 signaling pathway, inflammatory response, and KRAS signaling pathway. Angiogenesis is an important process in tumor metastasis and progression. In stomach cancer, angiogenesis could promote the tumor growth and metastasis (31). Fibroblasts, macrophages, and vascular endothelial growth factor are crucial factors in the angiogenesis and tumor progression of stomach cancer. As a conserved process in tumor cell genesis, epithelial–mesenchymal transition in stomach cancer could enhance tumor cell initiation, invasion, and chemoresistance. Hedgehog signaling could activate tumor cell proliferation by regulating the cell cycle in stomach cancer development and neoplastic transformation. The IL6-JAK-STAT3 signaling pathway is dysregulated in stomach cancer. Hyper-activation of the IL6-JAK-STAT3 signaling pathway is related with poor prognosis survival of stomach cancer patients. It facilitates the proliferation, invasion, and growth of tumor cells. The KRAS signaling pathway promotes metastasis and chemotherapy resistance of cancer stem cells in stomach cancer.

## Conclusion

This study found that a high expression of GPX8 in stomach adenocarcinoma was correlated with poor prognosis. Moreover, a high expression of GPX8 might exacerbate the stomach adenocarcinoma by enhancing the inflammation of the tumor microenvironment or taking part in several signaling pathways. Our study indicated that GPX8 was an important factor, which might be a potential target in the treatment of stomach adenocarcinoma.

## Data Availability Statement

The original contributions presented in the study are included in the article/**Supplementary Material**. Further inquiries can be directed to the corresponding author.

## Ethics Statement

The studies involving human participants were reviewed and approved by the Cancer Hospital, Chinese Academy of Medical Sciences, and Peking Union Medical College. The patients/participants provided their written informed consent to participate in this study.

## Author Contributions

XZ, HX, and YZ performed statistical analysis, and drafted the manuscript. CS, ZL, CH, and DZ contributed to database building. CG conceived the design of the study and revised the manuscript. All authors contributed to the article and approved the submitted version.

## Funding

This study was supported by grant from the Chinese Academy of Medical Sciences for CAMS Innovation Fund for Medical Sciences (CIFMS) (22021-I2M-1-061). The funders played no role in designing the study, collecting and analyzing data, deciding to publish, or preparing the manuscript.

## Conflict of Interest

The authors declare that the research was conducted in the absence of any commercial or financial relationships that could be construed as a potential conflict of interest.

## Publisher’s Note

All claims expressed in this article are solely those of the authors and do not necessarily represent those of their affiliated organizations, or those of the publisher, the editors and the reviewers. Any product that may be evaluated in this article, or claim that may be made by its manufacturer, is not guaranteed or endorsed by the publisher.

## Abbreviations

BLCA, bladder urothelial carcinoma; BRCA, breast invasive carcinoma; CHOL, cholangiocarcinoma; COAD, colon adenocarcinoma; ESCA, esophageal carcinoma; GBM, glioblastoma multiforme; HNSC, head and neck squamous cell carcinoma; KICH, kidney chromophobe; KIRC, kidney renal clear cell carcinoma; KIRP, kidney renal papillary cell carcinoma; LIHC, liver hepatocellular carcinoma; LUAD, lung adenocarcinoma; LUSC, lung squamous cell carcinoma; PAAD, pancreatic adenocarcinoma; PCPG, pheochromocytoma and paraganglioma; PRAD, prostate adenocarcinoma; READ, rectum adenocarcinoma; STAD, stomach adenocarcinoma; THCA, thyroid carcinoma; UCEC, uterine corpus endometrial carcinoma.
