# Supplementary material for: Immunohistochemistry and Bioinformatics Identify GPX8 as a Potential Prognostic Biomarker and Target in Human Gastric Cancer
Source: Front Oncol. 2022 May 27;12:878546. doi: 10.3389/fonc.2022.878546 (PMC9195577; doi:10.3389/fonc.2022.878546)
Supplement: Supplementary file 1 [file DataSheet_1.docx]

**Table S1：The association between GPX8 expression and clinicopathological variables**

| Characteristic | Low expression of GPX8 | High expression of GPX8 | p |
| --- | --- | --- | --- |
| n | 187 | 188 |  |
| T stage, n (%) |  |  | **< 0.001** |
| T1 | 18 (4.9%) | 1 (0.3%) |  |
| T2 | 45 (12.3%) | 35 (9.5%) |  |
| T3 | 79 (21.5%) | 89 (24.3%) |  |
| T4 | 45 (12.3%) | 55 (15%) |  |
| N stage, n (%) |  |  | 0.113 |
| N0 | 67 (18.8%) | 44 (12.3%) |  |
| N1 | 44 (12.3%) | 53 (14.8%) |  |
| N2 | 37 (10.4%) | 38 (10.6%) |  |
| N3 | 34 (9.5%) | 40 (11.2%) |  |
| M stage, n (%) |  |  | 0.230 |
| M0 | 167 (47%) | 163 (45.9%) |  |
| M1 | 9 (2.5%) | 16 (4.5%) |  |
| Pathologic stage, n (%) |  |  | **< 0.001** |
| Stage I | 40 (11.4%) | 13 (3.7%) |  |
| Stage II | 53 (15.1%) | 58 (16.5%) |  |
| Stage III | 74 (21%) | 76 (21.6%) |  |
| Stage IV | 13 (3.7%) | 25 (7.1%) |  |
| Race, n (%) |  |  | 0.050 |
| Asian | 40 (12.4%) | 34 (10.5%) |  |
| Black or African American | 9 (2.8%) | 2 (0.6%) |  |
| White | 111 (34.4%) | 127 (39.3%) |  |
| Gender, n (%) |  |  | 1.000 |
| Female | 67 (17.9%) | 67 (17.9%) |  |
| Male | 120 (32%) | 121 (32.3%) |  |
| Age, n (%) |  |  | 0.270 |
| <=65 | 76 (20.5%) | 88 (23.7%) |  |
| >65 | 109 (29.4%) | 98 (26.4%) |  |
| Histological type, n (%) |  |  | **< 0.001** |
| Diffuse Type | 23 (6.1%) | 40 (10.7%) |  |
| Mucinous Type | 4 (1.1%) | 15 (4%) |  |
| Not Otherwise Specified | 106 (28.3%) | 101 (27%) |  |
| Papillary Type | 4 (1.1%) | 1 (0.3%) |  |
| Signet Ring Type | 4 (1.1%) | 7 (1.9%) |  |
| Tubular Type | 46 (12.3%) | 23 (6.1%) |  |
| Residual tumor, n (%) |  |  | 0.058 |
| R0 | 163 (49.5%) | 135 (41%) |  |
| R1 | 5 (1.5%) | 10 (3%) |  |
| R2 | 5 (1.5%) | 11 (3.3%) |  |
| Histologic grade, n (%) |  |  | 0.195 |
| G1 | 5 (1.4%) | 5 (1.4%) |  |
| G2 | 77 (21%) | 60 (16.4%) |  |
| G3 | 101 (27.6%) | 118 (32.2%) |  |
| OS event, n (%) |  |  | **0.022** |
| Alive | 125 (33.3%) | 103 (27.5%) |  |
| Dead | 62 (16.5%) | 85 (22.7%) |  |
| PFI event, n (%) |  |  | 0.107 |
| Alive | 133 (35.5%) | 118 (31.5%) |  |
| Dead | 54 (14.4%) | 70 (18.7%) |  |
| DSS event, n (%) |  |  | 0.068 |
| Alive | 141 (39.8%) | 122 (34.5%) |  |
| Dead | 38 (10.7%) | 53 (15%) |  |
| Age, meidan (IQR) | 68 (59, 74) | 66 (57, 72) | 0.167 |

**Table S2: GPX8 expression association with clinical pathological characteristics (logistic regression)**

| Characteristics | Total(N) | Odds Ratio(OR) | P value |
| --- | --- | --- | --- |
| T stage (T3&T4 vs. T1&T2) | 367 | 2.032 (1.270-3.290) | 0.003 |
| N stage (N1&N2&N3 vs. N0) | 357 | 1.735 (1.103-2.747) | 0.018 |
| M stage (M1 vs. M0) | 355 | 1.821 (0.798-4.414) | 0.164 |
| Pathologic stage (Stage III&Stage IV&Stage II vs. Stage I) | 352 | 3.495 (1.841-7.040) | <0.001 |
| Gender (Male vs. Female) | 375 | 1.008 (0.661-1.539) | 0.969 |
| Residual tumor (R2 vs. R0&R1) | 329 | 2.549 (0.905-8.247) | 0.090 |
| Histologic grade (G3 vs. G1&G2) | 366 | 1.474 (0.969-2.248) | 0.070 |
| Age (>65 vs. <=65) | 371 | 0.776 (0.514-1.170) | 0.227 |

**Table S3:** Correlation analysis between GPX8 and the marker protein of immune cells

| Description | Gene markers | Cor | *P* | Description | Gene markers | Cor | *P* |
| --- | --- | --- | --- | --- | --- | --- | --- |
| T cell (general) | CD3D | 0.094 | 6.90E-02 | Dendritic cell | HLA-DPB1 | 0.17 | 9.17E-04 |
|  | CD3E | 0.095 | 6.57E-02 |  | HLA-DQB1 | 0.064 | 2.15E-01 |
|  | CD2 | 0.165 | 1.26E-03 |  | HLA-DRA | 0.113 | 2.77E-02 |
| CTL (Cytotoxic T Lymphocytes ) | CD8A | 0.121 | 1.88E-02 |  | HLA-DPA1 | 1.131 | 1.06E-02 |
|  | CD8B | 0.034 | 5.07E-01 |  | DEC-205 (LY75) | -0.064 | 2.15E-01 |
|  | GZMB | 0.003 | 9.46-01 |  | BDCA-1 (CD1C ) | 0.245 | 1.36E-06 |
| B cell | CD19 | 0.05 | 3.36E-01 |  | BDCA-4 (NRP1) | 0.536 | 1.58E-29 |
|  | CD79A | 0.116 | 2.34E-02 |  | BDCA-2 (CLEC4C) | 0.204 | 6.18E-05 |
|  | CD79B | 0.195 | 1.33E-04 |  | CD11c (ITGAX) | 0.281 | 2.52E-08 |
|  | CD22 | 0.18 | 4.18E-04 | Th1 | CD38 | 0.122 | 1.73E-02 |
| M1 Macrophage | INOS | -0.192 | 1.64E-04 |  | T-bet (TBX21) | 0.082 | 1.11E-01 |
|  | CIITA | -0.033 | 5.28E-01 |  | STAT4 | 0.169 | 9.57E-04 |
|  | IRF5 | 0.182 | 3.72E-04 |  | STAT1 | -0.057 | 2.68E-01 |
|  | COX2 (PTGS2) | 0.265 | 1.65E-07 |  | IFNG | 0.005 | 9.18E-01 |
| M2 Macrophage | CD163 | 0.332 | 3.35E-11 |  | TNF-ɑ (TNF) | 0.058 | 2.58E-01 |
|  | IRF4 | 0.102 | 4.62E-02 | Th2 | GATA3 | 0.241 | 2.15E-06 |
|  | VSIG4 | 0.409 | 9.40E-17 |  | IL13 | 0.114 | 2.70E-02 |
|  | MS4A4A | 0.426 | 3.57E-18 |  | STAT6 | -0.038 | 4.64E-01 |
| TAM | CCL2 | 0.41 | 8.03E-17 | Tfh | BCL6 | 0.325 | 8.80E-11 |
|  | CCL5 | 0.153 | 2.81E-03 |  | CD200 | 0.433 | 9.24E-19 |
|  | CD68 | 0.143 | 5.43-03 |  | IL21 | 0.048 | 3.49E-01 |
|  | IL10 | 0.341 | 9.10E-12 |  | ICOS | 0.053 | 3.05E-01 |
| Neutrophils | CD66b (CEACAM8) | -0.034 | 5.15E-01 | Th17 | STAT3 | 0.247 | 1.13E-06 |
|  | CD15 (FUT4) | -0.157 | 2.22E-03 |  | IL17A | -0.203 | 6.85E-05 |
|  | CD11b (ITGAM) | 0.36 | 5.17E-13 |  | IL1A | -0.005 | 9.20E-01 |
|  | CCR7 | 0.117 | 2.33E-02 |  | IL1B | -0.03 | 5.56E-01 |
| Natural killer cell | NKp46 (NCR1) | 0.072 | 1.65E-01 |  | CCL20 | -0.243 | 1.62E-06 |
|  | NKp44 (NCR2) | -0.022 | 6.73E-01 | Treg | FOXP3 | 0.061 | 2.35E-01 |
|  | NKp30 (NCR3) | 0.139 | 6.74-03 |  | CCR8 | 0.157 | 2.23E-03 |
|  | FCGR3A | 0.365 | 2.34-13 |  | TGFβ | 0.402 | 3.78E-16 |
|  | FCGR3B | 0.102 | 4.64E-02 | Immune Checkpoint | PD-1 | -0.02 | 6.92E-01 |
|  | NKG2A (KLRC1) | 0.185 | 2.94E-04 |  | CTLA4 | -0.043 | 4.03E-01 |
|  | KIR2DL1 | 0.123 | 1.68E-02 |  | TIM-3 | 0.333 | 2.98E-11 |
|  | KIR2DL3 | 0.041 | 4.28E-01 |  | PD-L1 | 0.045 | 3.87E-01 |
|  | KIR3DL1 | 0.082 | 1.11-01 |  | PD-L2 | 0.402 | 3.66E-15 |

**
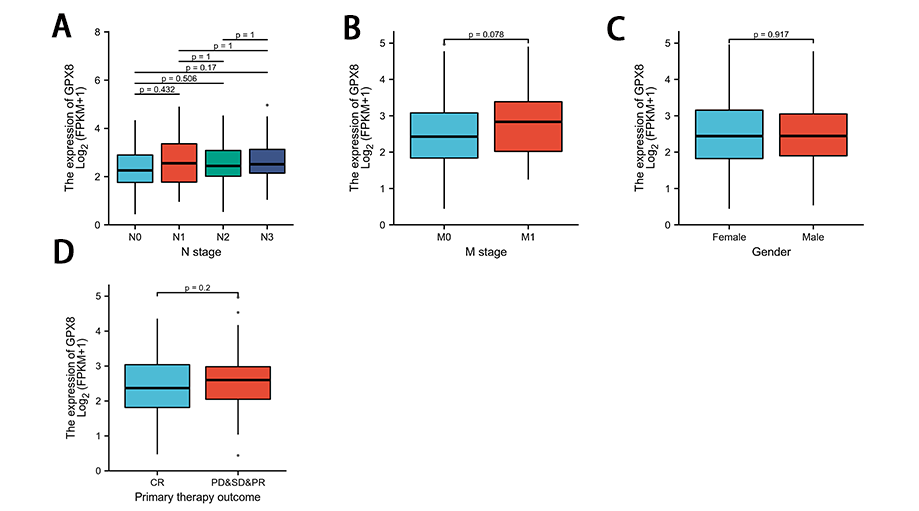
**

Figure S1: Association with GPX8 expression and clinicopathological characteristics, including. (A) N stage, (B) M stage, (C) Gender in stomach cancer patients in TCGA cohort and (D) Primary therapy outcome,.
